# Cholecystogastric fistula masquerading as gastrointestinal stromal tumour: Unusual presentation of gallbladder adenomyomatosis – a case report

**DOI:** 10.1097/MD.0000000000050819

**Published:** 2026-10-02

**Authors:** Gerelt-Od Khenmedekh, Dae Hoon Kim, Dong Hee Ryu, Kwon Cheol Yoo, Seung-Myoung Son

**Affiliations:** a Department of Surgery, Chungbuk National University College of Medicine, Cheongju, Republic of Korea; b Department of Surgery, Chungbuk National University Hospital, Cheongju, Republic of Korea; c Department of Pathology, Chungbuk National University College of Medicine, Cheongju, Republic of Korea; d Department of Pathology, Chungbuk National University Hospital, Cheongju, Republic of Korea.

**Keywords:** case report, cholecystogastric fistula, gallbladder adenomyomatosis, gastrointestinal stromal tumor, rokitansky-aschoff sinuses

## Abstract

**Rationale:**

We present the case of gallbladder adenomyomatosis (GA)-induced cholecystogastric fistula (CGF) that mimicked an invasive gastric gastrointestinal stromal tumor (GIST), leading to diagnostic and surgical challenges in an emergency setting.

**Patient concerns:**

A 71-year-old woman presented to the emergency department with acute abdominal pain and clinical signs of mechanical small-bowel obstruction (SBO).

**Diagnoses:**

Contrast-enhanced abdominal computed tomography (CT) revealed a 6-cm mass along the gastric antrum with apparent hepatic invasion and concurrent features of SBO. The gallbladder was not visualized. Esophagogastroduodenoscopy (EGD) demonstrated a subepithelial tumor-like bulge with a small mucosal opening in the gastric antrum, raising suspicion of a fistulous tract.

**Interventions:**

Emergency exploratory laparotomy identified two phytobezoars in the small bowel, which were treated by segmental resection and primary anastomosis. The gastric mass was found to be densely adherent to the liver, and bile leakage was encountered during dissection, prompting intraoperative hepatobiliary consultation.

**Outcomes:**

Histopathological examination of the resected specimen confirmed GA with inflammatory granulation tissue at the gallbladder–gastric serosal interface, with no evidence of malignancy. The patient recovered uneventfully and was discharged 29 days postoperatively.

**Lessons:**

This case highlights a novel and clinically significant diagnostic pitfall: GA-induced CGF can convincingly simulate an invasive gastric neoplasm on multimodal imaging and endoscopy. Recognition of biliary red flags, including non-visualization of the gallbladder on CT, a mucosal opening adjacent to a subepithelial-appearing lesion on EGD, and intramural cystic foci on imaging, is essential for accurate preoperative diagnosis.

## 1. Introduction

Cholecystogastric fistula (CGF) is an uncommon subtype of bilioenteric fistula, estimated to account for approximately 2% to 5% of all cholecystoenteric communications.^[1,2]^ The most common form of bilioenteric fistula is cholecystoduodenal fistula, followed by cholecystocolonic fistula, whereas gastric involvement is distinctly rare, reflecting the relative infrequency of sustained anatomical apposition between the gallbladder fundus and the gastric wall.^[1–3]^ When present, CGF typically arises as a late sequela of chronic cholecystitis and gallstone disease, in which recurrent cycles of inflammation, ischemia, and pressure necrosis lead to transmural erosion and fistula formation between the gallbladder and the adjacent viscus.

Gallbladder adenomyomatosis (GA) is a benign, noninflammatory hyperplastic condition of the gallbladder wall, characterized by epithelial invaginations into a hypertrophied muscularis propria, forming intramural diverticula known as Rokitansky–Aschoff sinuses (RAS).^[3–5]^ GA has been reported in up to 9% of cholecystectomy specimens and is broadly classified into 3 morphological subtypes: fundal (localized), segmental (annular), and diffuse.^[3,5]^ Although GA is generally considered a benign incidental finding, the localized fundal type may present as focal gallbladder wall thickening and has been recognized to radiologically mimic malignancies, including gallbladder carcinoma, or tumors of adjacent organs such as the stomach and colon.^[6–8]^ Despite its capacity to produce significant wall thickening and inflammatory changes, GA has not previously been implicated as a direct cause of CGF.

Gastrointestinal stromal tumors (GISTs) are the most common mesenchymal tumors of the gastrointestinal tract, originating from the interstitial cells of Cajal in the gastric muscularis propria. In contrast-enhanced computed tomography (CT), gastric GISTs characteristically appear as well-defined exophytic masses with avid arterial enhancement and, in larger lesions, central necrosis or hemorrhage.^[9,10]^

We herein present the case of CGF caused by GA, in which the biliary-origin inflammatory mass convincingly mimicked an invasive gastric GIST on preoperative CT and EGD, compounding diagnostic difficulty in the context of concurrent bezoar-induced small bowel obstruction (SBO) and a significant language barrier. This case provides a unique opportunity to characterize the diagnostic and surgical challenges of this rare entity and to propose a systematic CT-based diagnostic framework for gastric antral masses with suspected hepatic invasion.

## 2. Case presentation

A 71-year-old woman presented to the emergency department with a 7-day history of severe abdominal pain and distension that had not responded to conservative management at a local hospital. Her medical history was notable for type 2 diabetes mellitus and hypertension. Comprehensive history-taking was significantly impaired by a language barrier, as the patient was not a native Korean speaker. Laboratory investigations revealed mild renal impairment (creatinine 1.3 mg/dL) with otherwise normal complete blood count and liver function tests, including normal total bilirubin, alkaline phosphatase, and gamma-glutamyl transferase.

Contrast-enhanced abdominal CT demonstrated that a 6-cm mass-like lesion was identified along the gastric antrum, exhibiting wall thickening, abnormal mucosal enhancement, and apparent contiguity with the adjacent hepatic parenchyma, raising clinical suspicion for an invasive gastric –malignancy – specifically GIST with hepatic invasion (Fig. 1A), while it also marked dilation of the small-bowel loop consistent with mechanical SBO (Fig. 1B). Concurrently, the gallbladder was not visualized on CT, and in the absence of a reliable surgical history, this finding was initially attributed to prior cholecystectomy. No ectopic calcified stone was identified within the bowel lumen on CT.

**Figure 1.**
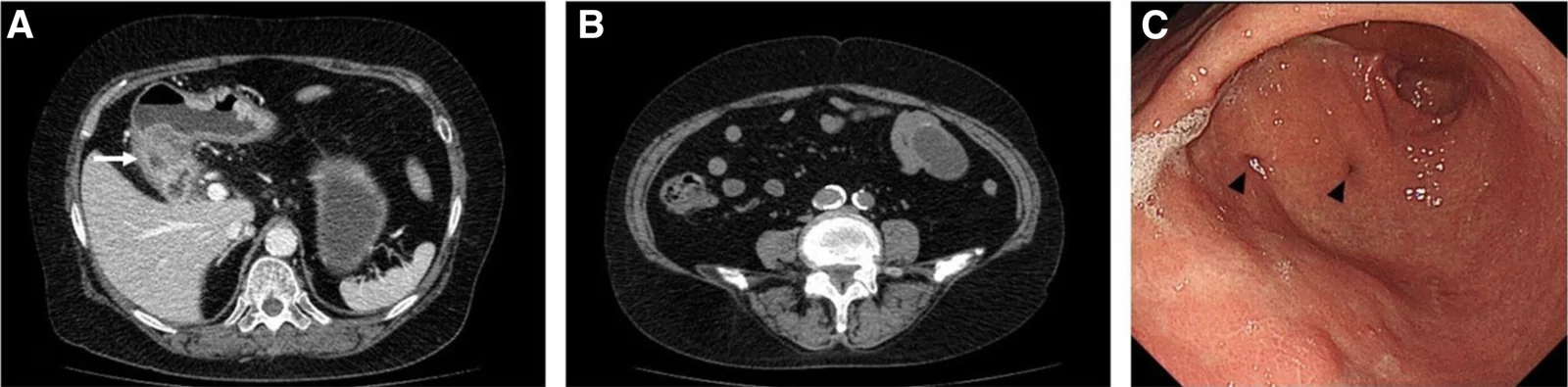
**Preoperative radiologic and endoscopic findings.** A and B. Contrast-enhanced abdominal CT demonstrates a 6-cm mass-like lesion along the gastric antrum with apparent contiguity with the adjacent hepatic parenchyma (white arrow), raising initial suspicion for invasive gastric GIST with hepatic extension. The gallbladder is not visualized. Concurrently, markedly dilated small-bowel loops (B) are consistent with mechanical SBO. C. Upper EGD reveals a subepithelial tumor-like bulge in the gastric antrum with thin overlying mucosa and prominent submucosal vascularity. A small mucosal opening (black arrowhead) is identified adjacent to the lesion, raising suspicion for a fistulous tract, although the defect was insufficient for direct endoscopic confirmation.

Upper EGD was performed and revealed a subepithelial tumor-like bulge in the gastric antrum with thin overlying mucosa and prominent submucosal vascularity, morphologically consistent with a subepithelial tumor (SET). A small mucosal opening was identified adjacent to the lesion (Fig. 1C); however, the defect was insufficiently large for direct endoscopic confirmation of a fistulous tract, and the acuity of the patient’s clinical presentation precluded further endoscopic maneuvers.

Given the clinical urgency of mechanical SBO with hemodynamic stability, emergency exploratory laparotomy was performed. Intraoperatively, two phytobezoar masses measuring 2 and 4 cm in diameter were identified in the small bowel lumen, located 90 and 200 cm distal to the ligament of Treitz, respectively. Segmental small-bowel resection with primary anastomosis was performed for both obstructing lesions.

A 6-cm firm mass was identified in the gastric antrum, with dense inflammatory adhesions to the adjacent hepatic parenchyma, visually and tactilely resembling an invasive GIST with direct hepatic extension. During dissection, unexpected bile leakage was encountered at the gastric–hepatic interface, fundamentally altering the intraoperative diagnostic impression and prompting urgent hepatobiliary (HBP) consultation. The HBP surgeon performed careful adhesiolysis and limited liver wedge resection of the adherent hepatic parenchyma. Progressive exploration of the mass revealed its biliary origin: the lesion represented an inflamed, fibrotic gallbladder adherent to and fistulizing into the posterior gastric antrum. Given the operative findings and continuing suspicion of neoplasia at the time of intraoperative decision-making, a subtotal gastrectomy was performed en bloc with the mass. A Jackson–Pratt drain was placed in the lesser sac.

The postoperative course was complicated by fever on postoperative day 5, prompting CT evaluation, which revealed a fluid collection and abscess formation at the hepatic bed and cholecystectomy site. The patient was managed with intravenous broad-spectrum antibiotics and percutaneous image-guided catheter drainage of the collection, resulting in gradual clinical improvement. She was ultimately discharged on postoperative day 29. She attended a postoperative outpatient follow-up visit on postoperative day 139; however, subsequently returned to her home country and was therefore unable to continue follow-up at our institution (Fig. 2).

**Figure 2.**
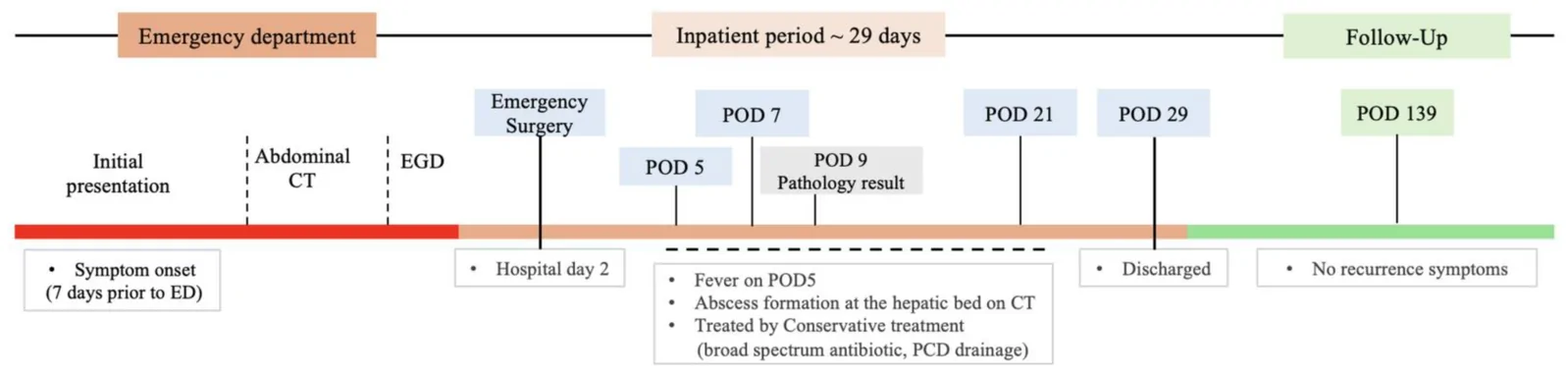
**Clinical timeline of the case.** Symptom onset preceded ED presentation by 7 days. Surgery was performed on HD 2 (POD 0). A postoperative complication (hepatic bed abscess) was managed conservatively from POD 5 to POD 21. The patient was discharged on POD 29 and followed up on POD 139 (~4.5 months post-operatively) without evidence of recurrence. HD, hospital day; POD, postoperative day; ED, emergency department; CT, computed tomography; EGD, esophagogastroduodenoscopy; PCD, percutaneous catheter drainage.

Histopathological examination of the resected specimen demonstrated GA, characterized by smooth muscle hyperplasia and intramural proliferation of RAS (Fig. 3A). At the gallbladder–gastric serosal interface, chronic inflammation and granulation tissue formation were identified, consistent with a fistulous tract of inflammatory rather than neoplastic origin (Fig. 3B). No dysplasia or malignancy was identified in any specimen component, definitively excluding an invasive gastric or biliary neoplasm.

**Figure 3.**
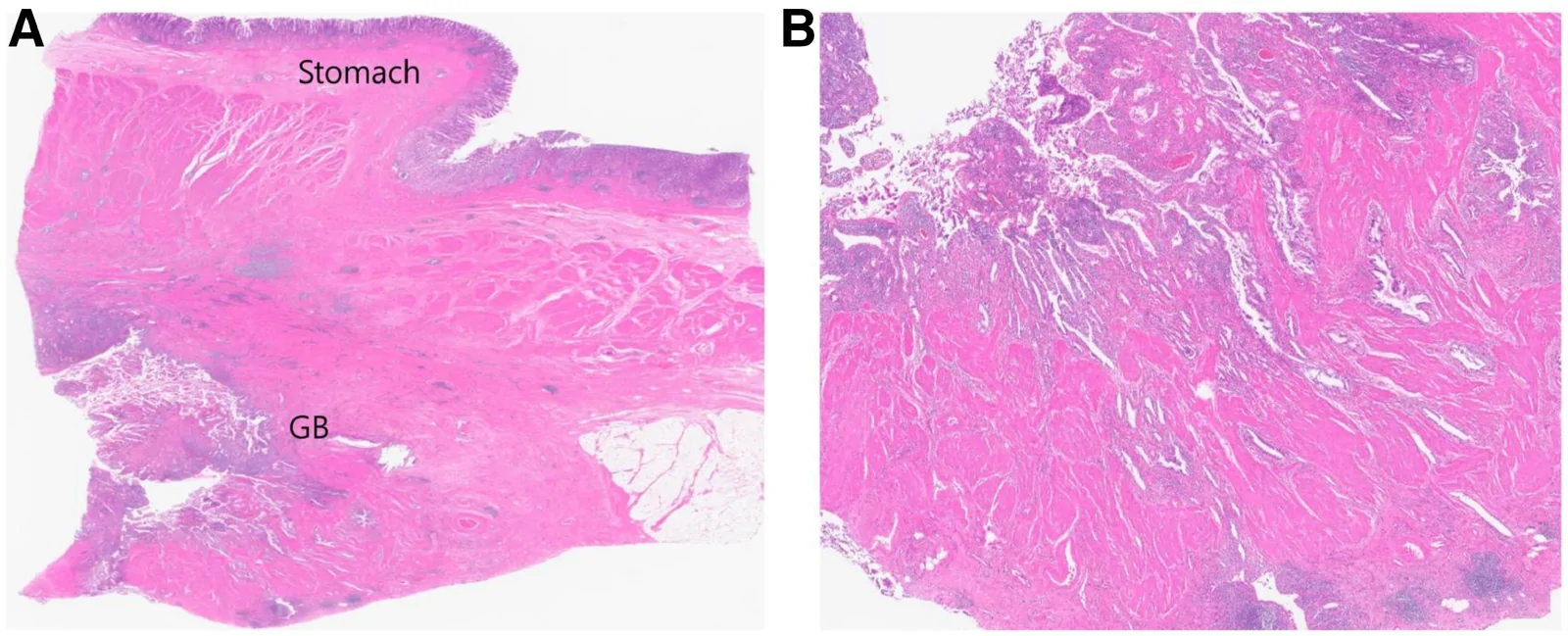
**Histopathological findings of the resected specimen.** (A) Low-power view (H&E, ×40) demonstrating adhesion between the gallbladder wall and the serosal surface of the stomach, with intervening chronic inflammation and granulation tissue formation at the fistulous interface. (B) Higher-magnification view (H&E, ×220; scale bar = 700 μm) of the gallbladder wall, showing intramural proliferation of Rokitansky–Aschoff sinuses (RAS) and smooth muscle hyperplasia, consistent with gallbladder adenomyomatosis. No dysplasia or malignancy was identified.

## 3. Discussion

### 3.1. Epidemiology and mechanisms of cholecystoenteric fistula

Cholecystoenteric fistulas (CEFs) are an uncommon but clinically significant complication of chronic gallbladder disease, occurring in an estimated 0.06% to 0.14% of patients undergoing cholecystectomy and in 0.1% to 0.5% of autopsy series.^[11,12]^ The pathogenesis invariably involves repeated cycles of acute-on-chronic cholecystitis, gallbladder distension, ischemia, and pressure necrosis that promote transmural erosion of the gallbladder wall into an intimately adherent viscus. The resulting fistula permits bidirectional passage of bile, bacteria, and – in a proportion of cases – migrated gallstones.

The distribution of CEF subtypes is anatomically predictable. Cholecystoduodenal fistulas are the most frequent, accounting for 60% to 85% of cases, owing to the consistent proximity of the gallbladder infundibulum to the first and second portions of the duodenum.^[2,11]^ Cholecystocolonic fistulas (involving the hepatic flexure or transverse colon) represent the second most common variant (10–25%), followed by cholecystogastric fistulas (2–5%) and, rarely, cholecystoileal or choledochoduodenal communications.^[1–3]^ The rarity of CGF reflects the anatomical reality that the gallbladder fundus is typically separated from the gastric body and antrum by intervening omentum and mesentery; sustained, direct gallbladder–gastric apposition sufficient to drive fistulization is uncommon.

In the present case, the underlying gallbladder pathology was GA rather than the more common simple cholecystitis or cholelithiasis. GA is a well-recognized benign condition, but its propensity for fistula formation has not been widely appreciated. We propose that in severe or recurrent forms of GA, particularly the segmental type, progressive smooth muscle hypertrophy and RAS-related wall remodeling may generate elevated intraluminal pressure, localized ischemia, and impaired mucosal barrier function – collectively creating conditions that facilitate transmural erosion and fistulization, even in the absence of frank gallstones. The case reported by Mann et al^[13]^ of a cholecystobronchial fistula secondary to GA represents the sole prior published case of GA-induced CEF in the English-language literature, further underscoring the novelty of the present report.

### 3.2. Imaging characteristics and the GIST mimicry

We also summarized the diagnostic characteristics and the GIST mimicry in Table 1. Gastric GISTs arise from the muscularis propria and typically present on contrast-enhanced CT as well-defined, exophytic masses with avid arterial enhancement. Larger tumors (>5 cm) frequently exhibit central necrosis, hemorrhage, or cystic degeneration. A pathognomonic, albeit uncommon, CT sign of gastric GIST is the presence of air or oral contrast material within an intratumoral cavity, reflecting mucosal ulceration and fistulization.^[10,14]^ The diagnostic characteristics and features distinguishing GIST from its mimics are also summarized in Table 1.

**Table 1:** Comparison of computed tomography (CT) and esophagogastroduodenoscopy (EGD) characteristics among Gastrointestinal Stromal Tumor (GIST), Gallbladder Adenomyomatosis (GA), and Cholecystogastric Fistula (CGF).

Feature	Gastric GIST	Gallbladder Adenomyomatosis (GA)	Cholecystogastric Fistula (CGF)
Computed tomography imaging features			
** Origin/primary pathology**	Gastric muscularis propria (4th EUS layer)	Gallbladder wall – epithelial hyperplasia	Gallbladder adherent to gastric antrum/body
	Mesenchymal tumor	Hypertrophy of muscularis propria	Chronic cholecystitis → transmural erosion
	Intact overlying gastric mucosa	Intramural diverticula (Rokitansky-Aschoff sinuses)	Fistulous communication between GB and stomach
** CT wall/ mass characteristics**	Well-defined exophytic submucosal mass	Focal/ segmental/ diffuse GB wall thickening	Thick-walled contracted/shrunken gallbladder
	Smooth serosal contour; intact mucosa	No discrete mass; no invasion of adjacent structures	Ill-defined wall between GB and gastric antrum
	Pedunculated endoluminal or dumbbell morphology	Hourglass deformity (annular type)	Slight deficiency/ discontinuity of GB wall at adherence
** CT enhancement pattern**	Avid arterial; ~98 HU on PVP	Intense mucosal epithelial enhancement (cotton-ball sign)	Thick-walled GB with mucosal enhancement
	Homogeneous if small; peripheral rim if large	Non-enhancing intramural cystic spaces (RAS)	Variable – reflects underlying chronic cholecystitis
	Heterogeneous with central necrosis > 5 cm	No mass enhancement; no pericholecystic stranding	Pericholecystic fat stranding from inflammation
** Pathognomonic/ hallmark CT sign**	*Air/ contrast in mass cavity – fistula*	*Rosary sign – CT*	*Pneumobilia (air in biliary tree*)
	Mucosal ulceration in ~50% gastric GIST	Intramural hypoattenuating cystic foci (RAS)	Linear gas-filled tract GB → gastric antrum
	Misdiagnosed as pancreatic pseudocyst on CT	Surrounded by unenhanced hypertrophied muscle	Pneumo-gallbladder or air-fluid level in GB
** Gallbladder on CT**	Normal gallbladder – not involved in gastric GIST	Thickened GB wall (focal/ segmental/ diffuse)	Shrunken, contracted, thick-walled GB
	No biliary communication	Gallstones in 90% segmental; ~50% diffuse/fundal types	GB adherent to gastric antrum – fat plane lost
	Gallstones not associated	RAS appear as hypoattenuating intramural foci	Calcified gallstones typically present (chronic cholelithiasis)
** Calcification**	Rare ~3%; posttreatment (imatinib)	Calcified stones/ cholesterol crystals within RAS	Calcified gallstones – radiopaque ectopic stone
		Porcelain GB wall in some cases	Stone may migrate into stomach (Bouveret variant)
** Necrosis/ cystic change**	Common in large tumors (> 5 cm); central fluid	RAS = cystic spaces in wall – diagnostic feature	Abscess formation in complicated chronic cholecystitis
	Central fluid in 67% malignant GIST (Burkill)	Not true necrosis; bile/ cholesterol/ sludge in sinuses	No central necrosis in uncomplicated CGF
	Haemorrhage → fluid-fluid level in liver mets	CEUS: RAS appear avascular	Empyema of GB if infected fistula
** Adjacent structure involvement**	No invasion; exophytic – compresses adjacent organs	No invasion; no pericholecystic fat stranding	Direct GB-to-stomach adherence/ wall fusion
	Hematogenous spread; no direct invasion	No adjacent structure involvement if uncomplicated	Ill-defined fat plane between GB and gastric antrum
	Peritoneal seeding only with rupture	Benign – does not transgress serosa	May involve duodenum, colon in other bilioenteric fistulas
** Gallstone/ background**	No gallstones; normal biliary tree	Gallstones very common (90% segmental type)	Gallstones mandatory – chronic cholelithiasis is cause
	Normal stomach and bowel	Background chronic cholecystitis	Large gallstone ≥ 2.5 cm typical (enables fistula)
			Bile reflux into stomach (bilious vomiting)

Endoscopic features			
** EGD gross appearance**	Submucosal bulge with intact overlying mucosa	Normal EGD – gallbladder not visible on EGD	*Fistulous opening in gastric antrum*
	Pillow sign (indent with biopsy forceps)	No direct EGD findings (extragastric pathology)	Small mucosal defect/ depression in posterior antrum
	Mobile, smooth-covered submucosal mass	Indirect: bile in stomach lumen if CGF co-exists	Active bile or pus drainage through opening
** Mucosal findings on EGD**	Intact, stretched mucosa over mass; ~50% ulcerated	No direct mucosal finding on EGD	Mucosal depression/ erythema at fistula site
	Bridging folds; pillow sign	EGD normal unless concurrent pathology	Bile outflow/ pus through fistula opening
	Surface biopsy often negative (intact mucosa)	Indirect: bile-stained gastric pool if bile reflux	Incarcerated gallstone may be visible in severe cases
** EGD biopsy/ therapeutic role**	Surface biopsy nondiagnostic (intact mucosa)	EGD not directly diagnostic for GA	EGD both diagnostic AND therapeutic in CGF
	EUS-FNA with CD117 IHC required for diagnosis	EUS may demonstrate GB wall thickening	Identifies fistula opening; allows stone extraction
	EGD useful for ulcer detection + haemostasis	No biopsy targets visible on EGD	Stent placement possible for large fistula orifice
** Associated EGD findings**	Normal surrounding gastric mucosa	Normal gastric mucosa on EGD	Bile reflux gastritis (bile-stained gastric pool)
	No bile reflux; no biliary communication	No direct EGD stigmata of adenomyomatosis	Pyloric stenosis if stone impacted (Bouveret)
			Gastric outlet obstruction signs: dilated stomach

CGF = cholecystogastric fistula, CT = computed tomography, EGD = esophagogastroduodenoscopy, EUS = endoscopic ultrasound, GA = gallbladder adenomyomatosis, GB = gallbladder, GIST = gastrointestinal stromal tumor, HU = Hounsfield unit, RAS = Rokitansky-Aschoff sinuses.

In the present case, the CGF-associated inflammatory mass convincingly reproduced several CT features of GIST: a 6-cm antral mass with abnormal mucosal enhancement and soft-tissue density; apparent contiguity with – and perceived invasion of – the adjacent hepatic parenchyma; and non-visualization of the gallbladder, which was erroneously attributed to prior cholecystectomy rather than prompting consideration of a collapsed or fistulized gallbladder. Two specific imaging features, had they been systematically sought, might have guided a different preoperative interpretation.

First, the gallbladder wall in GA characteristically demonstrates intramural hypoattenuating cystic foci (RAS) on CT, forming the so-called rosary sign, and intense mucosal epithelial enhancement producing a cotton-ball sign.^[15,16]^ Systematic interrogation of the pericholecystic region on CT – even in the perceived absence of the gallbladder – may have revealed wall stratification or residual intramural cysts consistent with GA. Second, non-visualization of the gallbladder on CT, in the absence of a confirmed prior cholecystectomy, constitutes an important biliary red flag that warrants active exclusion of a collapsed or fistulized gallbladder rather than reflexive attribution to surgical history.

On EGD, gastric GISTs classically present as subepithelial bulges with intact overlying mucosa, demonstrating the pillow sign (indentation with biopsy forceps) and occasional surface ulceration in approximately 50% of cases.^[17]^ In the present case, the endoscopic appearance of a subepithelial bulge with thin mucosa and prominent submucosal vascularity was identical, with the critical distinguishing feature being the small mucosal opening in the gastric antrum – a finding consistent with a fistulous communication rather than simple GIST-related mucosal erosion. Although the opening was too small for endoscopic confirmation in this emergency setting, recognition of this finding as a potential fistula red flag is essential: any minute mucosal defect adjacent to a subepithelial-appearing lesion should prompt active consideration of an extrinsic or inflammatory process before attributing it to a submucosal neoplasm.

Had the patient been hemodynamically stable, endoscopic ultrasound (EUS) would represent the optimal next diagnostic step: EUS enables precise characterization of the originating wall layer (muscularis propria for GIST vs extrinsic inflammatory adhesion for CGF) and can identify thickened gallbladder wall with intramural cystic spaces consistent with GA even when CT is nondiagnostic.^[18]^

### 3.3. Role of bezoar-induced sbo and the language barrier in diagnostic complexity

The diagnostic complexity of this case was substantially compounded by two concurrent factors: bezoar-induced SBO and a significant language barrier. Bezoar-related SBO, while uncommon overall, is disproportionately prevalent in patients with diabetes mellitus and prior gastric surgery, as both conditions impair gastric motility and promote intraluminal concretion formation.^[19–21]^ In patients with diabetes mellitus – as in the present case – delayed gastric emptying (gastroparesis) facilitates bezoar accumulation and increases the risk of downstream small-bowel impaction.

The clinical urgency imposed by mechanical SBO appropriately directed the initial management toward emergency surgery; however, it simultaneously limited the time available for detailed preoperative imaging review and multidisciplinary consultation. Had the clinical scenario permitted, MRCP or focused high-resolution CT of the pericholecystic region might have delineated the fistulous tract between the gallbladder fundus and the gastric antrum, potentially altering the preoperative diagnosis. This case therefore highlights the importance of maintaining a dual diagnostic index of suspicion in emergency abdominal surgery: while the primary indication for laparotomy may be unambiguous (SBO), the discovery of an unexpected ancillary mass warrants a systematic intraoperative approach before definitive resection.

The language barrier, which prevented reliable clarification of the patient’s surgical history, directly contributed to the misinterpretation of gallbladder non-visualization on CT. This underscores a broader principle in emergency surgical care: when accurate history cannot be obtained, the default assumption should be the more cautious interpretation, and the absence of a visible gallbladder should trigger consideration of pathological (rather than surgical) causes.

### 3.4. Surgical decision-making and the case for organ-preserving management

In this case, subtotal gastrectomy was performed intraoperatively based on the presumptive diagnosis of invasive GIST, a decision informed by the dense hepatic adhesions, palpable firmness of the mass, and intraoperative concern for malignancy. Ultimately, histopathology confirmed a benign inflammatory process, rendering gastrectomy unnecessary. This outcome exemplifies the clinical consequence of diagnostic mimicry: a benign, potentially organ-preserving condition prompted major visceral resection with its attendant morbidity, including a 29-day hospital stay complicated by a hepatic bed abscess requiring percutaneous drainage.

The standard definitive treatment for CGF is laparoscopic or open cholecystectomy with fistula takedown and primary repair of the enteric defect.^[2,11,12]^ When the gastric defect is small or accessible, primary closure in 2 layers (mucosa + seromuscular) is generally sufficient, whereas larger defects may require omental patching or, in rare circumstances, formal gastrectomy. Liver wedge resection, as performed in this case to address the adherent hepatic parenchyma, represents appropriate management when hepatic involvement cannot be excluded intraoperatively.

Had a CGF of biliary origin been suspected preoperatively, the surgical approach could have been planned as a targeted procedure: cholecystectomy, fistula excision, primary gastric repair, and selective liver wedge resection if the hepatic bed was involved – without subtotal gastrectomy. This organ-preserving approach would likely have reduced operative time, postoperative morbidity, and hospital stay.

### 3.5. Key teaching points

- Gallbladder adenomyomatosis, although typically benign, can cause cholecystogastric fistula through chronic inflammation-driven transmural erosion.
- A gastric antral subepithelial tumor-like mass with apparent hepatic invasion is not always neoplastic; biliary-origin inflammatory fistulization can faithfully reproduce the CT and EGD features of invasive GIST.
- Non-visualization of the gallbladder on CT is a critical biliary red flag that should prompt active exclusion of pathological (fistulized, collapsed) gallbladder rather than reflexive attribution to prior cholecystectomy, particularly when the surgical history is unclear.
- A small mucosal opening adjacent to a gastric subepithelial-appearing lesion on EGD should raise immediate suspicion of a fistulous process; systematic EUS assessment of the wall layer of origin is the recommended next step when feasible.
- Cholecystoenteric fistulas most commonly involve the duodenum (60–85%), followed by the colon (10–25%); gastric involvement (2–5%) is distinctly rare and reflects the relative anatomical remoteness of the gallbladder from the gastric wall.

In emergency abdominal surgery, the identification of an unexpected intraoperative finding (bile leakage, anatomical inconsistency with presumed diagnosis) should prompt immediate subspecialty consultation before definitive resection.

### 3.6. Limitations

Several limitations of the present report merit acknowledgment. First, this is a single-case report, and the generalizability of the proposed diagnostic framework requires validation in larger series. The extreme rarity of GA-induced CGF renders prospective study inherently challenging. Second, preoperative EUS was not performed due to the clinical urgency imposed by SBO; EUS may have provided critical information regarding the wall layer of origin of the mass and the presence of intramural RAS. Third, MRCP was not available as an adjunct preoperative investigation in the emergency setting. Fourth, the language barrier impeding history-taking is a largely contextual limitation; its impact on diagnostic accuracy, while significant in this case, may not be reproducible in other clinical environments. Finally, the retrospective recognition of imaging features consistent with GA (intramural cystic foci, wall stratification) on the original CT underscores the importance of systematic, structured CT reporting rather than pattern-recognition-based diagnosis.

## 4. Conclusion

We report the case of CGF arising from GA, in which the biliary-origin inflammatory mass convincingly simulated an invasive gastric GIST on CT and EGD, leading to diagnostic and surgical complexity in an emergency setting complicated by concurrent bezoar-induced SBO and a language barrier. Recognition of biliary red flags on CT (non-visualization of the gallbladder, intramural cystic foci, pneumobilia) and endoscopy (fistulous mucosal opening) is critical for accurate preoperative diagnosis.

## Author Contributions

**Data curation:** Gerelt-Od Khenmedekh, Dae Hoon Kim, Seung-Myoung Son.

**Writing – original draft:** Gerelt-Od Khenmedekh, Dae Hoon Kim.

**Conceptualization:** Dae Hoon Kim.

**Supervision:** Dae Hoon Kim, Dong Hee Ryu, Seung-Myoung Son.

**Writing – Review & Editing:** Dae Hoon Kim, Dong Hee Ryu, Kwon Cheol Yoo.
